# Supplementary material for: Identification and functional analysis of non-coding regulatory small RNA FenSr3 in Bacillus amyloliquefaciens LPB-18
Source: PeerJ. 2023 May 15;11:e15236. doi: 10.7717/peerj.15236 (PMC10194069; doi:10.7717/peerj.15236)
Supplement: Supplemental Information 4 [file peerj-11-15236-s004.zip › KO/CK-vs-T1_map/map04216.html]

KEGG PATHWAY: Ferroptosis - Reference pathway


|  |  |
| --- | --- |
| **Ferroptosis - Reference pathway** |  |

[
Pathway menu
| Organism menu
| Pathway entry
| Show description
| User data mapping
]

Ferroptosis is a regulated form of cell death and characterized by a production of reactive oxygen species (ROS) from accumulated iron and lipid peroxidation. It can be induced by experimental compounds (e.g.,erastin, RSL3) or clinical drugs(e.g., sulfasalazine, sorafenib) in cancer cell and certain normal cells. It is involved in multiple physiological and pathological processes, such as cancer cell death, neurodegenerative disease, tissue damage and acute renal failure.

|  |  |  |
| --- | --- | --- |
| Reference pathway | 184% 150% 122% 100% 82% 67% 55% | 图片下载 |
